# Understanding the Dynamics of the Structural States of Cannabinoid Receptors and the Role of Different Modulators

**DOI:** 10.3390/life12122137

**Published:** 2022-12-18

**Authors:** Anjela Manandhar, Mona H. Haron, Michael L. Klein, Khaled Elokely

**Affiliations:** 1 Institute for Computational Molecular Science and Department of Chemistry, Temple University, Philadelphia, PA 19122, USA; 2 National Center for Natural Products Research, University of Mississippi, Oxford, MS 38677, USA

**Keywords:** cannabinoid receptors, THC, Vitamin E, ionic lock, rotameric toggle switch

## Abstract

The cannabinoid receptors CB_1_R and CB_2_R are members of the G protein-coupled receptor (GPCR) family. These receptors have recently come to light as possible therapeutic targets for conditions affecting the central nervous system. However, because CB_1_R is known to have psychoactive side effects, its potential as a drug target is constrained. Therefore, targeting CB_2_R has become the primary focus of recent research. Using various molecular modeling studies, we analyzed the active, inactive, and intermediate states of both CBRs in this study. We conducted in-depth research on the binding properties of various groups of cannabinoid modulators, including agonists, antagonists, and inverse agonists, with all of the different conformational states of the CBRs. The binding effects of these modulators were studied on various CB structural features, including the movement of the transmembrane helices, the volume of the binding cavity, the internal fluids, and the important GPCR properties. Then, using in vitro experiments and computational modeling, we investigated how vitamin E functions as a lipid modulator to influence THC binding. This comparative examination of modulator binding to CBRs provides significant insight into the mechanisms of structural alterations and ligand affinity, which can directly help in the rational design of selective modulators that target either CB_1_R or CB_2_R.

## 1. Introduction

G protein-coupled receptors, also known as GPCRs, are the largest family of membrane proteins. They are made up of seven transmembrane helices (TM1 to TM7) that are connected by intracellular (IC) and extracellular (EC) loops. Because GPCRs are involved in important physiological processes, such as cell regulation, immunological responses, and signal transduction, they are one of the most important protein targets for the research into and development of new drugs. In fact, around one third of all currently available drugs are designed to affect GPCRs [1,2]. Cannabinoid receptors (CBRs) belong to Class A, the “rhodopsin-like family,” which is the largest subfamily of GPCRs. They are essential components of the endocannabinoid system [3].

Around three decades have passed since the identification of CBRs as the protein target of $\Delta^{9}$-tetrahydrocannabinol ($\Delta^{9}$-THC), the primary psychotropic ingredient of the cannabis plant [4,5]. The activation and inhibition of CBRs have been the focus of many studies ever since, because of their roles in a wide range of disorders, including those affecting sensation [6,7,8], memory [9], and appetite [10]. Cannabinoid Receptor 1 (CB_1_R) and Cannabinoid Receptor 2 (CB_2_R) are the two types of human cannabinoid receptors currently identified. These receptors are homologous, sharing 44% sequence identity; the primary variation between them is where in the body they are distributed [11]. The expression of CB_1_R is widespread throughout the body, with the highest levels found in the central nervous system (CNS). On the other hand, CB_2_R is largely found in the immune system, with lower levels identified in the CNS [12,13]. Several studies have pointed to the possibility that CB_1_R could play a role in the treatment of pain [14,15], anxiety [16,17], obesity [18,19], cancer [20,21,22], and neurodegenerative diseases [23,24,25]. In a similar vein, CB_2_R has been suggested to have a possible function in the regulation of pain [26,27], pruritus [28,29], neuropathy [30,31], and liver cirrhosis [32,33]. Therefore, CBRs have a significant degree of potential as drug targets for therapeutic use.

The activities of the CBRs are controlled by a pair of endogenous cannabinoids called 2-carachidonoyl glycerol (2-AG) and N-arachidonoylethanolamide (AEA, anandamide) [34]. Endocannabinoids such as anandamide and 2-AG bind to the cannabinoid receptors at the orthosteric site. In addition to naturally occurring cannabinoids and cannabinoids derived from plants, researchers are also working to develop synthetic cannabinoids that are more pharmacologically active. These ligands are placed into one of three categories, depending on their activity: agonist, antagonist, or inverse agonist. The paucity of the crystal structures of CBRs has been a major roadblock to structure-based drug development for many years. CBRs, like other GPCRs, are hardly expressed in recombinant hosts and are unstable in surfactants, making crystallization a challenge. Therefore, researchers have relied on homology models derived from the crystal structures of different GPCRs. In 2016, thanks to advances in GPCR crystallography, the crystal structures of CB_1_R in its inactive state when bound to the antagonist AM6538 and the inverse agonist taranabant were obtained [35,36]. A year later, the crystal structures of CB_1_R when bound to the agonists AM5112 and AM841 were determined, shedding light on the structural distinction between the active and inactive states of CB_1_R [37]. According to structural data for CB_1_R complexes, an agonist has a smaller ligand binding site and a more stable rotameric toggle switch between Phe200^3.36^ and Trp356^6.48^ than an antagonist does. In 2019, the first crystal structure of CB_2_R in its inactive state bound to the antagonist AM10257 was reported [38]. The study concluded that the size of the CB_2_R antagonist binding pocket is equivalent to the volume of the CB_1_R agonist binding pocket, implying structural similarity between the two. These crystal structures, which offered critical insight into the orthosteric/allosteric binding sites and the residues essential for ligand binding, have paved the way for future structural and dynamic studies of these systems.

Understanding the molecular interactions and subsequent conformational changes generated by ligand binding is critical for rational drug design. The mechanisms of ligand-receptor binding and receptor activation/inhibition have been successfully elucidated with the use of molecular dynamics (MD) simulations. Using systematic molecular modeling and simulation approaches such as homology modeling, docking, and all-atom MD simulations, we analyzed the active, inactive, and intermediate states of CB_1_R and CB_2_R. We modeled intermediate-state CB_1_R, active-state CB_2_R, and inactive-state CB_2_R based on the published crystal structures of CB_1_R and CB_2_R. Here, we have carefully studied the residues involved in ligand interaction and tracked the conformational changes of transmembrane helices upon ligand binding.

The Binding Database was used to collect the selected representative agonist, antagonist, and inverse agonist ligands. For each of the cannabinoid receptors CB_1_R and CB_2_R, we simulated 24 systems for 0.5–1 µs (18 µs total) as follows: agonist bound to active states, antagonist bound to inactive states, inverse agonist bound to active states, and all ligand types (agonist, antagonist, and inverse agonist) bound to intermediate states. To begin, we defined the ligand binding pocket and located the pivotal residues involved in ligand interaction. Next, we examined the differences in TM mobility across the various CBR conformations. We then examined the ionic lock between residues Arg^3.50^ and Arg^6.50^, as well as the rotameric toggle switch between residues Phe^3.36^ and Trp^6.48^, which are both known to characterize the active vs. inactive state of GPCRs. Next, we explored the significance of vitamin E as a lipid regulator of the cannabinoid system and its effect on the binding of the partial agonist, Δ^9^-THC. According to our earlier findings, vitamin E may reduce the binding of Δ^9^-THC to CB_2_R, either by forming adducts with Δ^9^-THC or by changing the conformation of the binding cavity [39]. The use of vitamin E acetate as a THC diluent has been linked to EVALI [40]. In this current study, we performed two additional MD simulations of CB_1_R active states with and without α vitamin Es to investigate the effect of vitamin Es on Δ^9^-THC binding.

## 2. Materials and Methods

### 2.1. Protein Preparation

From the Protein Data Bank, we obtained the crystal structures of active-state CB_1_R (PDB 5XR8, 5U09) [36,37], inactive-state CB_1_R (PDB 5TGZ) [35], and inactive-state CB_2_R (PDB 5ZTY) [38]. The crystal structures of the active state of CB_1_R in complex with the agonist AM841 and in complex with the inverse agonist taranabant were used. To aid crystallization, all structures had been mutated and joined with a stabilizing protein in ICL3. These mutations were reversed in our study, and the fusion proteins were deleted. The missing ICL3 segment was then rebuilt by crosslinking the two ends of ICL3 with the aid of the BioLuminate module of the Schrödinger suite [41,42,43,44]. The final structures were then achieved with the protein preparation wizard workflow [45]. AM841 and taranabant from the CB_1_R active states, AM6538 from the CB_1_R inactive state, and AM10257 from the CB_2_R inactive state were removed, as well as crystallization excipients and crystallographic water molecules. Then, at a pH of 7.4, the proper protonation and tautomerization states were assigned, hydrogen bond networks were optimized, and the resulting structures were energy minimized using the OPLS3e forcefield [46].

Prime was used to model the CB_2_R active state, as well as the intermediate states of CB_1_R and CB_2_R [47]. The prepared active CB_1_R structure in complex with AM841 was used to model the active CB_2_R structure. The constructed active CB_1_R structure and the closest rhodopsin protein’s intermediate state structure from the BLAST search were used to model the intermediate CB_1_R structure using a multiple template technique. The prepared intermediate CB_1_R structure was used as a template to model the intermediate CB_2_R structure. 

### 2.2. Ligand Preparation and Docking

Twelve selective ligands were selected from the BindingDB database, including two agonists [48,49,50,51], two antagonists [52,53,54,55], and two inverse agonists [56,57,58,59] for both CB_1_ and CB_2_ receptors. The structures and respective lists of ligands are provided in Table 1 and Table 2. Each ligand was then prepared for docking using LigPrep [60] with appropriate tautomers and stereoisomers assigned at a pH of 7.0 using Epik [61].

For docking, grid generation application of Glide was used to create an orthosteric site receptor grid for each structure prior to the docking experiment [62,63,64,65]. Each crystal structure was aligned with either its original crystal structure or the crystal structure from which the homology model was built, so that they shared the same reference frame. Receptor grids were constructed using information about bound ligands. Then, we docked the THC and the prepared ligand library using Glide’s SP (Standard precision) mode [62]. Five poses per ligand were generated for each docking iteration, and the one with the lowest score was chosen.

### 2.3. System Setup

For the simulations, a total of 24 structures were prepared, including the active-state CBR complex with two agonists, the active-state CBR complex with two inverse agonists, the inactive-state CBR complex with two antagonists, and the intermediate-state CBR complex with the six ligands. The DESMOND system builder module was utilized for the initial system configuration [66]. All CB structures were immersed in a POPC lipid bilayer, neutralized with NaCl ions, and dissolved in TIP3P water [67]. The positions of the CB structures in the membrane were determined using the OPM database [68]. The system details are provided in Tables S1–S4.

### 2.4. Vitamin E and THC System Setup

The THC was docked into the CB_1_R orthosteric binding site, and then two different MD simulation systems were built, one with five vitamin Es in the upper leaflet of the cell membrane surrounding active-state CB_1_, and the other without. Our prior work has covered the system setup in detail [39].

### 2.5. Molecular Dynamics Simulations

All MD simulations were run in the DESMOND system of the Schrödinger suite [66] using an OPLS3e force field [46]. The pressure was kept constant at 1 bar and the temperature was kept constant at 300° K, using the Nose–Hoover chain [69] and Martyna-Tobias-Klein coupling [70] schemes respectively. The RESPA integrator was used in the numerical integration with a short-range/bonded interaction updated every 2 ps and long-range/non-bonded interactions updated every 6 ps [71]. The short-range Coloumb interactions had a cutoff of 9.0 Å, and the long-range interactions were calculated using the particle mesh Ewald method, with a tolerance of 1 × 10^−9^ [72]. After minimization, each active and inactive CBR system was run for 1 μs and the intermediate CBR system was run for 500 ns, with the NPT ensemble trajectory being stored every 10 ps. Similarly, CB_1_R with α vitamin Es surrounding it and the CB_1_–THC complex were run for 1 μs and 200 ns respectively.

### 2.6. CB_1_R In Vitro Binding Assay

The affinities of THC for CB_1_R were examined using displacement assays, as previously described [39]. Briefly, cell membranes from CHO cells expressing human CB1Rs were isolated using differential centrifugation. THC in PG with and without vitamin E were incubated with the isolated membrane in a binding buffer (50 mM Tris-HCl, 1 mM EDTA, 3 mM MgCl2, 5 mg/mL BSA, pH 7.4) along with 2.5 nM [3H]CP-55,940. Total binding was assessed in the presence of an equal concentration of DMSO, while nonspecific binding was determined in the presence of 10 μM CP-55,940, and background binding was determined in wells lacking a membrane. Following incubation at 30 °C for 60 min, the binding reactions were terminated by filtration through Whatman GF/C filters. The filters were then washed twice with an ice-cold buffer (50 mM Tris-HCl, 1 mg/mL BSA). A liquid scintillation cocktail was added to each well, and the total tritiated counts per minute were analyzed using a TopCount scintillation counter. Background counts were subtracted from all wells and the percentage displacement from total binding was calculated. THC was screened at 4–250 μg/mL of PG concentrations alone or in the presence of 50% vitamin E acetate or vegetable glycerin. 

## 3. Results

### 3.1. Protein-Ligand Interaction Profile

#### 3.1.1. CB_1_R Active and Inactive States

**Figure 1:**
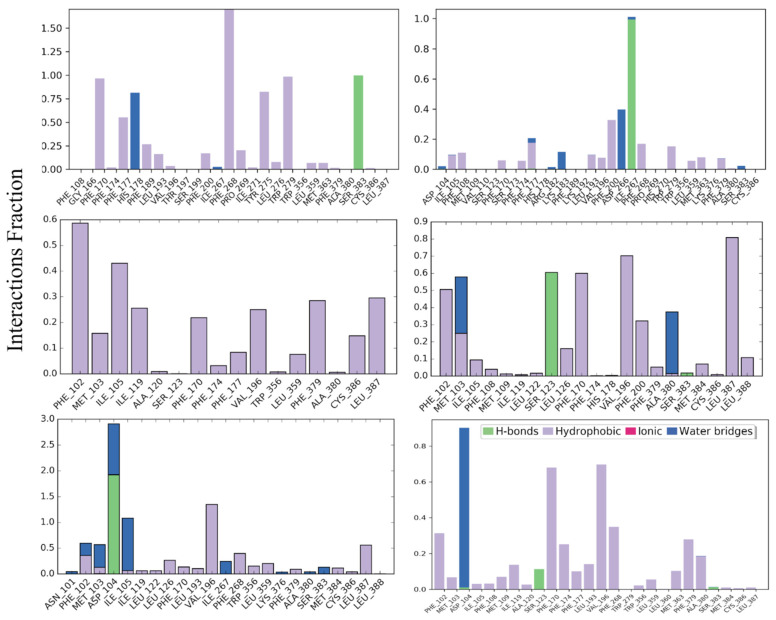
Interaction fractions of amino acid residues of active- and inactive-state CB_1_R with agonists, antagonists, and inverse agonists.

The protein-ligand interaction profile of each docked ligand with its corresponding CBR was then carefully analyzed for the last 100 ns of MD simulations. All CB_1_R structures demonstrated strong H-bond and hydrophobic interactions with their respective ligand, as illustrated in Figure 1. The most common ligand-interacting residues in the agonist-bound states were Phe^177^, Phe^268^, and Trp^279^; in antagonist-bound states were Phe^102^, Met^103^, Phe^170^, Val^196^, and Leu^387^; and in inverse agonist-bound states were Asp^104^ and Val^196^. 

#### 3.1.2. CB_1_R Intermediate States

In the CB_1_R intermediate states shown in Figure S1, Phe^379^ was a common residue that interacted strongly with all ligands. Other common residues interacting with ligands in agonist-bound states included Phe^200^ and Trp^279^; in antagonist-bound states included Phe^177^, Leu^193^, and Val^196^; and in inverse agonist-bound states included Phe^177^, Phe^189^, Leu^193^, Val^196^, and Pro^268^. When compared to the active and inactive CB_1_R states, the interactions that Phe^379^ had with the ligands were substantially stronger in the case of the CB_1_R intermediate states. Phe^379^ demonstrated multiple π−π interactions with the antagonists, and a single π−π interaction with the agonists and inverse agonists. It’s interesting to note that in CB_1_R intermediate states, most of the residues involved in ligand binding were those that come after position 165. As a result, residues comprising TM1 did not play an active role in ligand interaction in CB_1_R intermediate states.

#### 3.1.3. CB_2_R Active and Inactive States

For CB_2_R active and inactive states, ligand-residue interactions were different for different states and ligand type (Figure S2). The common ligand-interacting residues in agonist-bound states included Phe^94^, PHE^117^, Trp^194^ and Phe^281^; in antagonist-bound states were Phe^102^, Leu^170^, Val^196^, and Leu^387^. For inverse agonist bound complexes there were no common residues but Asp^104^, Val^196^, Tyr^25^, Met^26^, Phe^94^, His^95^, Phe^183^, and Phe^281^ were dominant residues.

#### 3.1.4. CB_2_R Intermediate States

In a similar manner, the most common residue engaged in ligand interactions for all CB_2_R intermediate states was Phe^281^ (Figure S3). Besides Phe^281^, other frequent ligand-interacting residues in the agonist-bound states included Ile^110^, whereas Phe^183^ was involved in antagonist-bound states, and Ile^110^ and Phe^117^ were involved in the inverse agonist-bound states. The interaction of Phe^183^ with ligands is absent for agonists and reduced for inverse agonists. When compared to the active and inactive states of CB_2_R, the interaction between Phe^183^ and antagonists is only significant in the CB_2_R intermediate state. Similarly, the interactions with Trp^194^ in CB_2_R intermediate states are only significant in the case of agonist 1 and are absent or reduced in other ligands.

In general, we detected different residues of the CBRs interacting with their respective ligands. Interestingly, Phe^379^ (CB_1_R) and Phe^281^ (CB_2_R) represent the conserved residue Phe^7.35^ (Ballesteros and Weinstein numbering [73,74]), and they had stronger interactions with ligands in the intermediate states of both CB_1_R and CB_2_R. 

### 3.2. Binding Cavities 

#### 3.2.1. Position of the Binding Cavity

**Figure 2:**
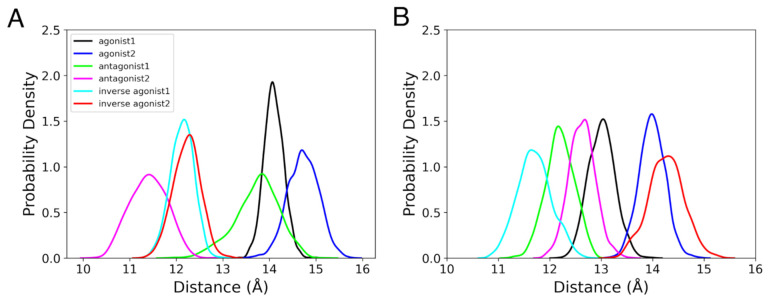
Probability densities of the distance between COMs of the ligands and active or inactive states of (**A**) CB_1_R and (**B**) CB_2_R for the last 100 ns MD simulations.

We estimated the location of the binding cavity by measuring the distance between the center of mass (COM) of the ligand and that of the CBR. In both the active and inactive stages of CBRs, the binding cavity was found to be mostly located between 10 Å and 16 Å from the COM of the receptor, based on the last 100 ns of the MD simulations (Figure 2). Remarkably, in CB_1_R intermediate states, the ligand was just 15–22 Å from the COM of the receptor, putting it closer to the extracellular region (Figure S4A). In CB_2_R intermediate states, the antagonist binding cavity was located 17–22 Å from the COM of the receptor, suggesting that the cavity is pushed upward compared to other ligand-bound intermediate conformations (Figure S4B).

#### 3.2.2. Volume of the Binding Cavity

**Figure 3:**
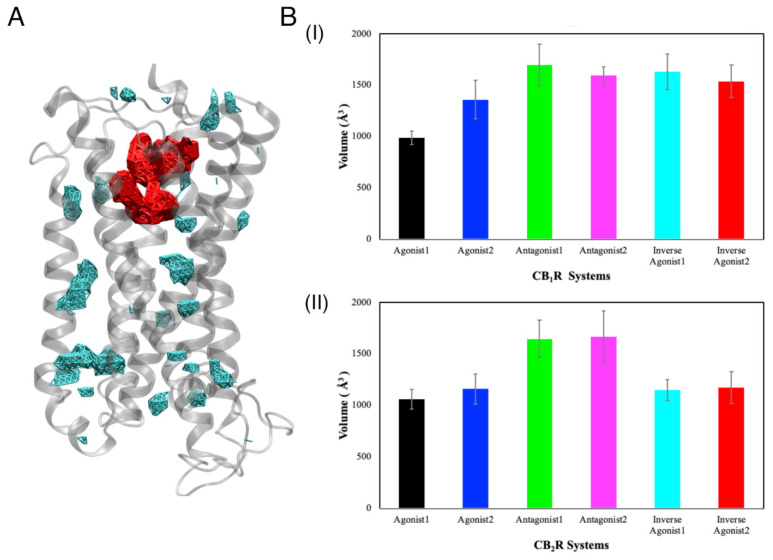
(**A**) A representation of the orthosteric binding cavity (red) and other cavities (cyan) in a CBR (gray). (**B**) The average volume of the orthosteric binding site cavity for different ligand-bound states of (**BI**) CB_1_R and (**BII**) CB_2_R, during 1 µs MD simulations.

Next, we used the Fpocket [75] to analyze the change in the volume of the ligand binding cavity over time (Figure S5). The volume of the orthosteric binding cavity was calculated for our systems and is shown in red (Figure 3A). For active and inactive CB_1_R states (Figure 3(BI)), the volume of the binding cavity was found to be significantly smaller in the case of the agonist 1 bound form (volume in presence of agonist 1 was 987.05 Å^3^ ± 64.78 Å^3^, agonist 2 was 1360.33 Å^3^ ± 187.89 Å^3^, antagonist 1 was 1696.19 Å^3^ ± 202.79 Å^3^, antagonist 2 was 1594.71 Å^3^ ± 84.11 Å^3^, inverse agonist 1 was 1631 Å^3^ ± 173.73 Å^3^, and inverse agonist 2 was 1539.02 Å^3^ ± 158.33 Å^3^). The binding cavities for antagonists and inverse agonists bound CB_1_R systems were larger. This difference can be seen in the crystal structures of CB_1_R, where the volume of the binding cavity associated with the agonist was reported to be ~384 Å^3^, and with the antagonist, as ~822 Å^3^ [35]. The study reported a 53% decrease in the volume of the CB_1_R ligand binding cavity in the case of the agonist-bound state compared to the antagonist-bound state. Our CB_1_R systems exhibited binding cavities with a volume that was double the value indicated but was consistent with crystal structures. The volume in the presence of agonist 1 was smaller than the volume in the cases of inverse agonists and antagonists. Our investigation of the transitional stages demonstrated no definite trend between the intermediate states (Figure S5B,D). For CB_1_R intermediate states, the volume of the binding cavity increased for the first 100 ns, before stabilizing between ~800–2100 Å^3^ for the last 100 ns. Here, the volume of the binding cavity was greatest for the antagonist- and smallest for the agonist-bound case. For CB_2_R active and inactive states, antagonist-bound systems had significantly larger volumes compared to agonist- and inverse agonist-bound systems (Figure 3(BII)). Similarly, the CB_2_R antagonist-1-bound system had a larger binding cavity compared to other ligand-bound systems (Figure S5D).

#### 3.2.3. Internal Waters 

**Figure 4:**
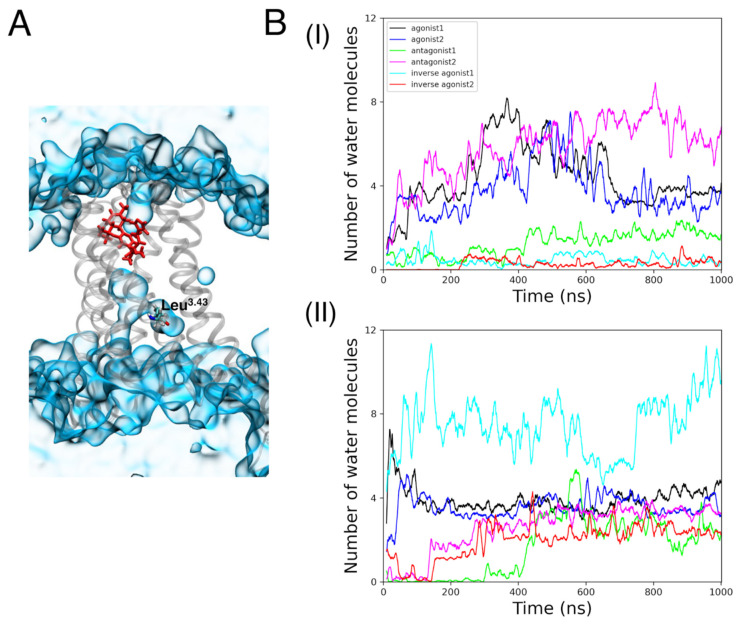
(**A**) A representation of internal water molecules (blue, quick surf representation) around Leu^3.43^ (licorice representation) in a CB receptor (gray). (**B**) The number of internal waters in (**BI**) CB_1_R active and inactive states (**BII**) CB_1_R active and inactive states, during the 1 µs MD simulations.

A previous study by Dror et al. [76] reported an increase in the number of water molecules in the cavity between TM3, TM5, TM6, and TM7 during the activation of a GPCR $\beta_{2}$-adrenergic receptor. In our study, the number of internal waters is defined as the number of oxygen atoms of the water molecule within 8 Å of Leu^3.43^. Water molecules, as illustrated in Figure 4A, were seen within the CBR binding cavity alongside the ligand and Leu^3.43^. 

In the CB1R agonist-bound complex, internal water molecule concentration increased for around 600 ns before decreasing (Figure 4B). It is remarkable that antagonist 2 retained the earlier trend of the agonist-bound complexes by having a rising number of internal waters. Throughout the 1 µs MD simulations, these three states (agonist 1, agonist 2, and antagonist 2) exhibited the highest number of internal water molecules in comparison to other states. For CB_2_R, inverse agonist 1 bound to the active state showed the greatest number of internal waters (about seven). Other active and inactive states of CB_2_R, at the conclusion of the MD simulations, had roughly three internal waters.

There was no appreciable buildup of internal waters during the MD simulations for CB_1_R intermediate states. Meanwhile, the number of internal waters fluctuated in the CB_2_R intermediate states with no clear trend (Figure S6).

### 3.3. Structural Properties

#### 3.3.1. Helix Conformational Analysis

**Figure 5:**
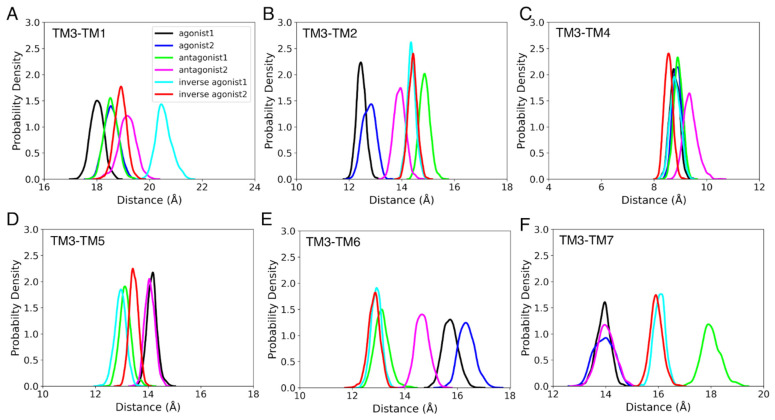
The probability density for the distance between COMs of TM3 and (**A**) TM1, (**B**) TM2, (**C**) TM4, (**D**) TM5, (**E**) TM6, and (**F**) TM7, during the last 100 ns of the 1 µs MD simulation of CB_1_R active and inactive states.

**Figure 6:**
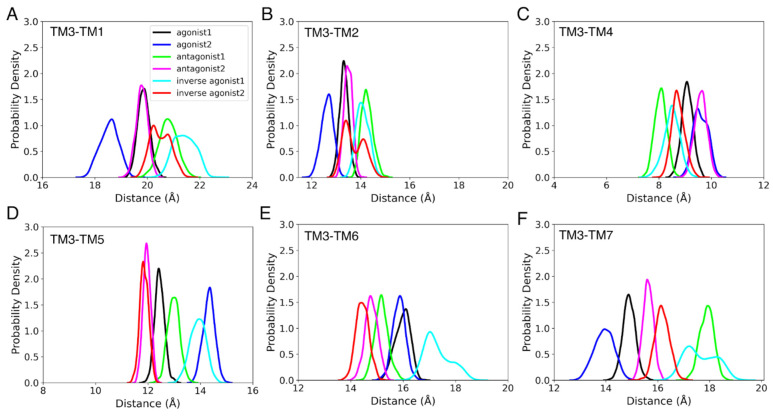
The probability density for the distance between COMs of TM3 and (**A**) TM1, (**B**) TM2, (**C**) TM4, (**D**) TM5, (**E**) TM6, and (**F**) TM7, during the last 100 ns of the 1 µs MD simulation of CB_2_R active (agonist and inverse agonist) and inactive (antagonist) states.

Rearranging the helices of a GPCR is a necessary part of the activation, particularly in the intracellular region [77]. There are reports of significant conformational changes occurring in TM3, TM5, TM6, and TM7 during GPCR activation. We measured the difference between the COMs of TM1, TM2, TM4, TM6, and TM7 with respect to the COM of TM3 to follow this rearrangement. The differences between the COMs of each helix and TM3 for the active and inactive states of CB_1_R and CB_2_R are depicted in Figure 5 and Figure 6. We discovered that in the agonist-bound states of CB_1_R, TM2 and TM7 are closest to TM3, while TM6 is farthest away. Intriguingly, the TM1 of the agonist-bound states is closest to the TM3 at the start of the MD simulations, but over time, the TM1 of the antagonist-bound states and the inverse agonist-bound states moved towards the TM3. Additionally, we observed that, with the movement of TM6 away from TM3 and the movement of TM7 closer to TM3, antagonist-2-bound CB_1_R switched to a state that was similar to an agonist-bound state. Only TM7, in the case of CB_2_R, displayed a distinct pattern, with the TM7 of agonist-bound states being closest to TM3. A similar analysis was also conducted for intermediate states, but no definite trend was found.

#### 3.3.2. Ionic Lock

**Figure 7:**
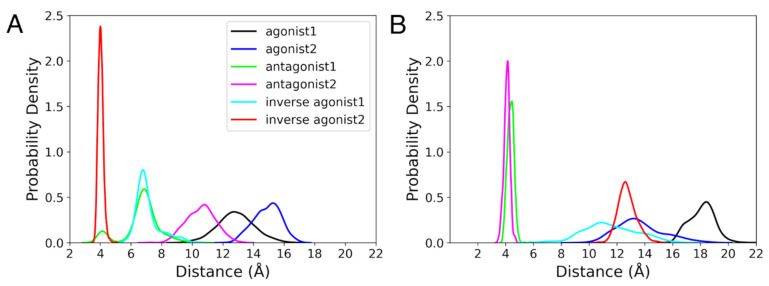
Ionic Lock. The probability density for the difference between COMs of Arg^3.50^ and Asp^6.30^ during the last 100 ns of the 1 µs MD simulation of the active and inactive states of (**A**) CB_1_R and (**B**) CB_2_R.

It is known that the salt bridge between Arg^3.50^ of the DR^3.50^Y motif of TM3 with Asp^6.30^ of TM6 exists in the inactive state of GPCRs [78]. This interaction is termed as the ionic lock, and it is broken in the active state. The ionic lock distance in the active state of the CB_1_R crystal structure is 14.2 Å and in the inactive state of the CB_1_R crystal structure is 6.7 Å [79]. In the instance of the agonist-bound states, the ionic lock broke at distances greater than 10 Å for CB_1_R (Figure 7A). The distance between Arg^3.50^ and Asp^6.30^ in the case of antagonist 2 was less than that of the agonist-bound states but mostly remained below 10 Å, which indicates that the ionic lock is broken. It was only for agonist 2 in complex with the CB1R intermediate states where the ionic lock was broken (Figure S7B). By the end of the MD simulations, the ionic lock distances for the CB_1_R intermediate states of agonist 1, antagonists, and inverse agonists were less than 7 Å. In CB_2_R active and inactive states, the ionic lock was broken in the case of the agonist and inverse agonist bound states, with a distance range greater than 10 Å (Figure 7B). In the meantime, only inverse agonist 2 of the CB_2_R intermediate states had a broken ionic lock, with a distance greater than 10 Å (Figure S7D). The orientations of Arg^3.50^ and Asp^6.30^ in the last frame of the MD simulations are shown in Figure S8. We noticed that Asp^6.30^ had changed its position, while Arg^3.50^ was roughly in the same location for all CBRs. The shift of Asp^6.30^ is correlated with the TM6 movement (discussed earlier).

#### 3.3.3. Rotameric Toggle Switch

**Figure 8:**
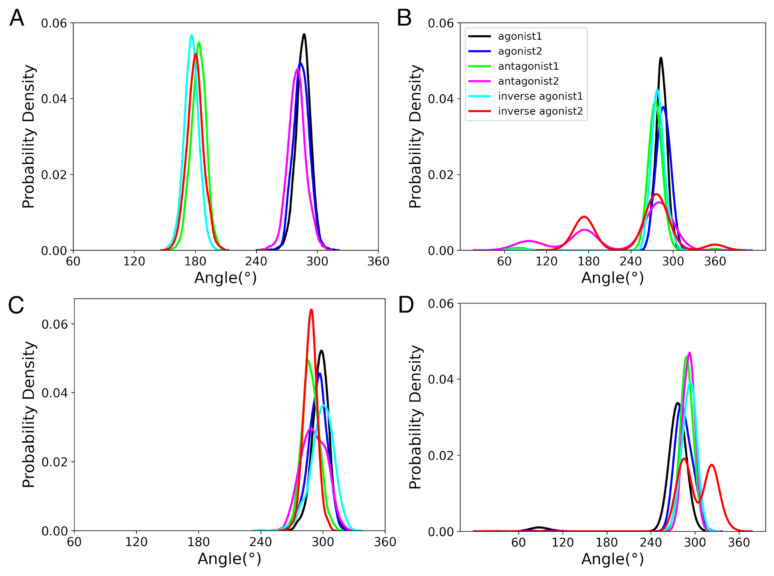
Rotameric switch. Probability density of the dihedral angles of (**A**) Phe^3.36^ in CB_1_R active and inactive states, (**B**) Phe^3.36^ in CB_2_R active and inactive states, (**C**) Trp^6.48^ in CB_1_R active and inactive states, and (**D**) Trp^6.48^ in CB_2_R active and inactive states, during 100 ns of the 1 µs MD simulation.

The dihedral angle (χ1) switch of Trp^6.48^ and Phe^3.36^ side chains is another element that has been found to differentiate between the active and inactive states of GPCRs. The rotameric toggle switch is reported to switch from trans to gauche (+) conformation for Phe^3.36^ and gauche (+) to trans conformation for Trp^6.48^ during the activation of GPCRs [80]. Trp^6.48^ and Phe^3.36^ preserve aromatic stacking in the inactive state, which is lost upon activation. The switch angles are classified as follows: 0° to 120° as gauche (−), 120° to 240° as trans, and 240° to 360° as gauche (+). Figure 8 shows the probability densities of χ1 of Trp^6.48^ and Phe^3.36^ during the last 100 ns of MD simulations of CBR active and inactive states. For CB_1_R active and inactive states, the χ1 value of Phe^3.36^ was in the trans conformation in the case of inverse agonists and antagonist-1-bound states, and in the gauche (+) conformation for the agonist and antagonist-2-bound states (Figure 8A) during last 100 ns of 1 µs MD simulation. Suggesting a change in activation state, χ1 of Phe^3.36^ of antagonist-2-bound states switched from an initial trans state to a gauche (+) state at ~380 ns (Figure S9A). There was no switch in χ1 values for the Trp^6.48^ of CB_1_R active and inactive states (Figure 8C). Phe^3.36^ maintained its trans conformation in CB_1_R intermediate states (Figure S9B). This implies that the intermediate CB_1_R states are more comparable to the CB_1_R inactive states. Similar to active and inactive CB_1_R systems, the χ1 value of Trp^6.48^ in CB_1_R intermediate states adopted gauche (+) conformation for the majority of the MD simulation time (Figure S9C,D). In CB_2_R active and inactive states, the χ1 value of Phe^3.36^ and Trp^6.48^ alternated between gauche (−) and gauche (+), (Figure S10A,C) but remained predominantly in the gauche (+) conformation during last 100 ns of 1 µs MD simulation (Figure 8B,D). In CB_2_R intermediate states, although the Phe^3.36^ of both antagonist states started in the gauche (+) conformation, the antagonist-1-bound state changed to trans at about 150 ns (Figure S10B). The Trp^6.48^ for CB_2_R intermediate states remained in the gauche (+) conformation throughout the simulation (Figure S10D). The orientations of Phe^3.36^ and Trp^6.48^ at the last MD simulation snapshot are shown in Figure S11. We found that the stacking of Phe^3.36^ and Trp^6.48^ was only maintained in the CB_1_R antagonist 1 and in the inverse agonist-bound states. Phe^3.36^ and Trp^6.48^ had a conformation that was comparable to that of the CB_1_R antagonist bound and inverse agonist bound states; however, there was no aromatic stacking between these residues.

### 3.4. Effect of Vitamin E on THC Binding

#### 3.4.1. Possible THC–CB_1_R Binding Modulation by Vitamin E Acetate In Vitro

**Figure 9:**
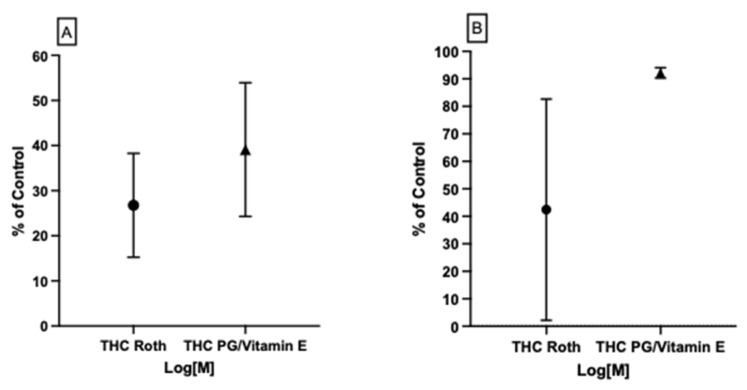
In vitro assessment of CB_1_R binding affinity to THC with and without vitamin E acetate for (**A**) lower and (**B**) higher THC concentrations. Up to 50% more displacement (less binding) of THC from CB_1_R was observed in the presence of vitamin E acetate in comparison to the pattern seen with THC in PG only.

Our previous work [39] found that vitamin E/acetate has the ability to modulate the binding affinity of CB2 to THC in vitro as well as in molecular docking models. In this study, we tested the possibility of vitamin E acetate having the same effect on CB_1_R binding affinity to THC in vitro. We tested two THC concentration ranges for a complete assessment of the vitamin E acetate effect on THC–CB_1_R binding. At 50% vitamin E acetate to 50% THC in propylene glycol (PG), volume-wise, the affinity of THC for CB_1_R was examined using a radio ligand displacement assay, as previously described. Our results showed ~12% more displacement (less binding) for THC at concentrations ranging from 0.041 μM to 10 μM in the presence of vitamin E acetate (Figure 9A). Meanwhile, at higher THC concentrations of 796 μM to 0.125 μM (250 μg/mL to 4 μg/mL), there was around 50% more THC displacement (less binding) in the presence of vitamin E acetate (Figure 9B). These current and previous results suggest that vitamin E/acetate can be a strong modulator of both CB_1_R and CB_2_R binding affinity to THC, and maybe to other cannabinoids. 

#### 3.4.2. MD Simulations of CB_1_ in the Presence and Absence of Vitamin E

**Figure 10:**
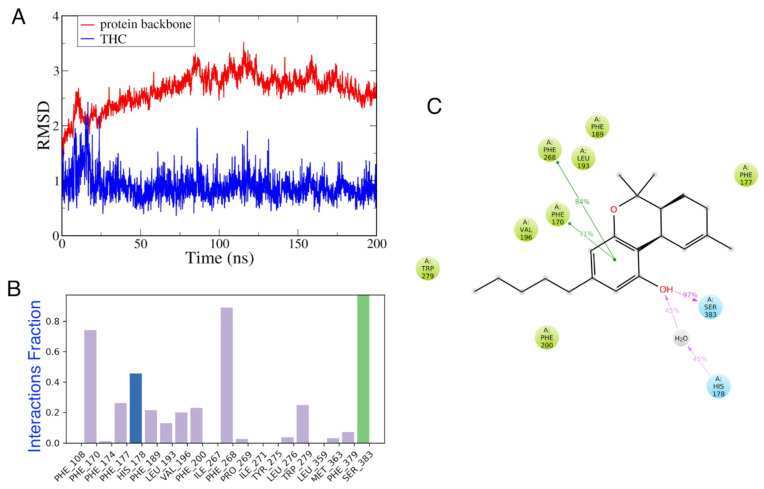
CB1–THC complex. (**A**) The RMSDs of CB1 backbone and THC, (**B**) interaction fraction, and (**C**) a 2D interaction diagram of THC interacting with CB_1_R.

In this study, we investigated how THC binds to CB1, and then how α-tocopherol affected that binding. Figure 10 demonstrates that after 50 ns, THC remained firmly bound to the CB_1_R. According to the results of the analysis of the interaction profile, THC formed strong H-bonds with Ser^383^, and interacted hydrophobically with Phe^268^, Phe^170^, Phe^177^, and Trp^279^. It also interacted with His^178^ via a bridged water molecule. At least 70% of the time during these MD simulations, the π−π interactions between THC and Phe^268^ or Phe^170^ were steady. When compared to the interaction profile of THC with CB_2_R, this pattern was consistent [39]. Moreover, the interaction pattern of THC observed here was similar to the agonist-like pose, as reported by *Dutta* et al., with the common residues—Leu^193^, Val^196^, Trp^279^, Ser^383^, and Phe^379^ interacting with THC [81].

After the 1 μs MD simulation of the CB_1_ receptor in the presence of vitamin Es, four clusters were generated. The relative binding energy was then determined using the Prime MM-GBSA method [82] after docking THCs to each cluster. Table 3 displays the results of a comparison between these figures and the final snapshot of the 200 ns CB1–THC system. These binding energy values were compared with that of the last frame at the 200 ns of the CB_1_–THC system, as shown in Table 3.

## 4. Discussion

Here, we modelled the active, inactive, and intermediate states of CBRs and investigated the structural changes upon the binding of different modulators –agonists, antagonists, and inverse agonists. We also investigated the effect of vitamin Es on THC binding to CB_1_R. The in vitro data confirm the data obtained through the molecular docking work, where they both demonstrated around 50% less THC–CB_1_R binding affinity in the presence of vitamin E. Based on our previous work [39] and this current work, THC–CB_1_R and CB_2_R activity would be significantly lower than expected for a given THC concentration. In the case of smoking or vaping THC with vitamin E acetate, this decrease in THC–CB_1_R activity could be a factor re-enforcing more smoking or vaping to compensate for the decreased psycho-effect of THC. On the other hand, vitamin E acetate can reduce THC-anti-inflammatory CB_2_R’s effectiveness and enhance a pro-inflammatory microenvironment [39]. An increase in the pro-inflammatory microenvironment in the lungs, in addition to more vaping or smoking to compensate for the decreased psycho-effect, may lead to significant lung inflammation and could explain how vitamin E acetate contributed to the 2019 EVALI outbreak.

Regarding the interaction profile, there was no clear trend with respect to the class of modulator or the state of CBR. However, there was a common residue, Phe^3.45^, which showed interesting behavior across different CBR states and modulator types. Phe^3.45^ showed strong ligand interaction in CB_1_R and CB_2_R intermediate states. While exploring the structural properties and comparing them with other GPCR properties, CB_1_R’s properties agreed more compared to CB_2_R’s. The properties of intermediate states were not significantly different either due to the modulator type or the CBR state. This is to be expected since the intermediate state can have either active-like or inactive-like conformation. However, the MD simulations here could not provide solid evidence for the conformational change towards an active-like state or inactive-like state upon the binding of agonists or antagonists, respectively. The interactions fraction, internal water molecules, and volume of the binding cavity provide insight into the dynamics of ligand CBR interaction in the binding cavity. The information regarding residues having significant ligand interactions and the binding pocket volume can be used to design novel active modulators.

Moving the focus beyond the binding cavity, additional properties, such as ionic lock, rotameric switch, and helix movements, were analyzed to understand the conformational changes upon ligand binding to CBRs. For the ionic lock, in agreement with the GPCR property, both CB_1_R and CB_2_R active states had broken the salt bridge between Arg^3.50^ and Asp^6.30^. For CB_1_R, one of the antagonists succeeded in breaking the salt bridge, suggesting a shift from an inactive to an active state. Interestingly, inverse agonist-bound states for CB_2_R also had broken salt bridges. For CB_1_R, the broken salt bridge in the active state correlates to the movement of TM6 away from TM3, as shown in Figure 7. The outward movement of TM6 is a typical property of GPCR activation [83]. Here, for CB_1_R, the crystal structure of inverse agonist-bound CB_1_R was available, and for CB_2_R, the modelled active-state CB_2_R was used as the target of inverse agonists. A longer MD simulation or enhanced sampling might provide alternative conformations of inverse agonist-bound CB_2_R state, which currently seems to be trapped in its original conformation. Another important feature of GPCRs is the rotameric toggle switch, a phenomenon during which the side chains of Trp^6.48^ and Phe^3.36^ undergo gauche-to-trans and trans-to-gauche transformations, respectively. Only the Phe^3.36^ of CB_1_R active and inactive states followed the trend, while Trp^6.48^ stayed in the gauche (+ve) conformation for the majority of the simulation of all systems. For CB_2_R active and inactive states, both Trp^6.48^ and Phe^3.36^ were mostly in gauche (+ve) states. For CB_2_R, fluctuating transformations were observed but were not as dominant as observed in CB_1_R. 

Overall, in this study we investigated the interaction pattern and structural changes a CBR can undergo in its active, inactive, or intermediate state. Using recent crystal structures and modelled structures of the CBRs, the structural properties, such as the *ionic lock* and the rotameric toggle switch, were compared with the established GPCR properties. Although the pattern did not agree exactly with GPCR properties, it shines a light on the necessity of additional modeling studies of CBRs to understand these systems better. To establish a better understanding of CBRs, further studies with additional modulators using multiple replicas, long-time scale simulations, or enhanced sampling can be conducted. For example, a recent study by Dutta et al., utilizing both active and inactive states of CB_1_R and CB_2_R, performed very long unbiased MD simulations (700 μs) and adaptive sampling to further characterize CBR states, ligand selectivity, and activation mechanism [84]. Besides different simulation approaches, cross-docking agonists to inactive state and antagonists to active state can reveal the switching of active, inactive states and the mechanism following it. 

## Figures and Tables

**Table 1:** The selected agonists, antagonists, and inverse agonists for CB_1_R.

BDBM50233600	BDBM50432728
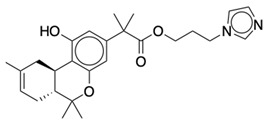	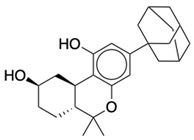
Agonist 1	Agonist 2
BDBM50195530	BDBM50399518
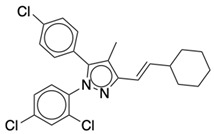	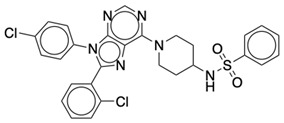
Antagonist 1	Antagonist 2
BDBM50198734	BDBM50198718
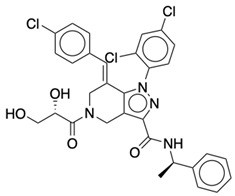	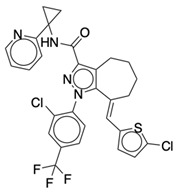
Inverse Agonist 1	Inverse Agonist 2

**Table 2:** The selected agonists, antagonists, and inverse agonists for CB_2_R.

BDBM50006259	BDBM50005278
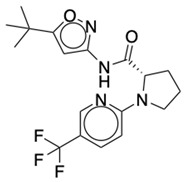	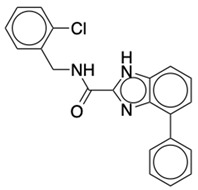
Agonist 1	Agonist 2
BDBM50116984	BDBM50180022
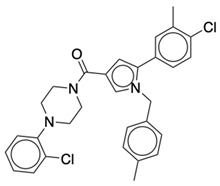	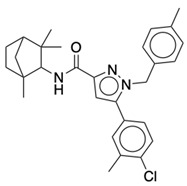
Antagonist 1	Antagonist 2
BDBM50420884	BDBM50154629
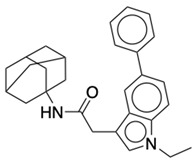	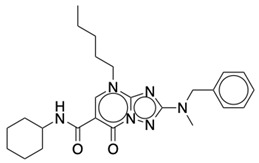
Inverse Agonist 1	Inverse Agonist 2

**Table 3:** Docking score and relative binding energies for THC–CB_1_R complexes in the presence and absence of α vitamin Es.

Systems		Docking Score	MM-GBSA ΔG_Bind_ (Kcal/mol)
CB1 with vitamin E	Cluster 1	−6.227	−46.60
	Cluster 2	−6.571	−49.22
	Cluster 3	−6.913	−10.72
	Cluster 4	−6.466	−46.57
CB1–THC complex (last frame)		−11.404	−86.45

## Data Availability

Initial.cms files of MD simulations of the 24 systems discussed in Tables S1–S4 and THC docked systems can be made available on request from the corresponding authors.

## Supplementary Materials

The following supporting information can be downloaded at: https://www.mdpi.com/article/10.3390/life12122137/s1, Figure S1. Interactions fraction of amino acid residues of intermediate CB1R states during last 100ns MD simulations with agonists, antagonists, and inverse agonists; Figure S2. Interactions fraction of amino acid residues active and inactive CB_2_R states during last 100 ns MD simulations with agonists, antagonists, and inverse agonists; Figure S3. Interactions fraction of amino acid residues of intermediate CB_2_R states with agonists, antagonists, and inverse agonists; Figure S4. Locations of orthosteric binding sites in (A) CB_1_R intermediate states and (B) CB_2_R intermediate states; Figure S5. The volume of the binding site cavity for different ligand-bound states of (A) CB_1_R active and inactive states (B) CB_1_R intermediate states, (C) CB_2_R active and inactive states, and (D) CB_2_R intermediate states; Figure S6. The number of internal waters in (A) CB_1_R and (B) CB_2_R intermediate states; Figure S7. Running average for the difference between COMs of Arg^3.50^ and Asp^6.30^ in (A) CB1 active and inactive states (B) CB1 intermediate states (C) CB2 active and inactive states and (D) CB2 intermediate states; Figure S8. Positions of amino acid residues Arg^3.50^ and Asp^6.30^ participating in ionic lock at the end of 500ns MD simulations for (A) CB_1_R regular states (B) CB_1_R intermediate states, (C) CB_2_R regular states, and (D) CB_2_R intermediate states; Figure S9. Dihedral angle of PHE^3.36^ in (A) CB_1_R active and inactive states and (B) CB_1_R intermediate states; and dihedral angle of Trp^6.48^ in (C) CB_1_R active and inactive states and (D) CB_1_R intermediate states; Figure S10. Dihedral angle of PHE^3.36^ in (A) CB_2_R active and inactive states and (B) CB_2_R intermediate states; and dihedral angle of Trp^6.48^ in (C) CB_2_R active and inactive states and (D) CB_2_R intermediate states; Figure S11. The orientations of Phe^3.36^ and Trp^6.48^ at the last MD simulation snapshot of CB_1_R and CB_2_R active, inactive, and intermediate states; Table S1. System details for the active and inactive states CB_1_R; Table S2. System details for the intermediate states CB_1_R; Table S3. System details for the active and inactive states CB_2_R; Table S4. System details for the intermediate states CB_2_R.
