# Supplementary material for: Understanding the Dynamics of the Structural States of Cannabinoid Receptors and the Role of Different Modulators
Source: Life (Basel). 2022 Dec 18;12(12):2137. doi: 10.3390/life12122137 (PMC9786085; doi:10.3390/life12122137)
Supplement: Supplementary file 1 [file life-12-02137-s001.zip › life-1965075-supplementary.pdf]

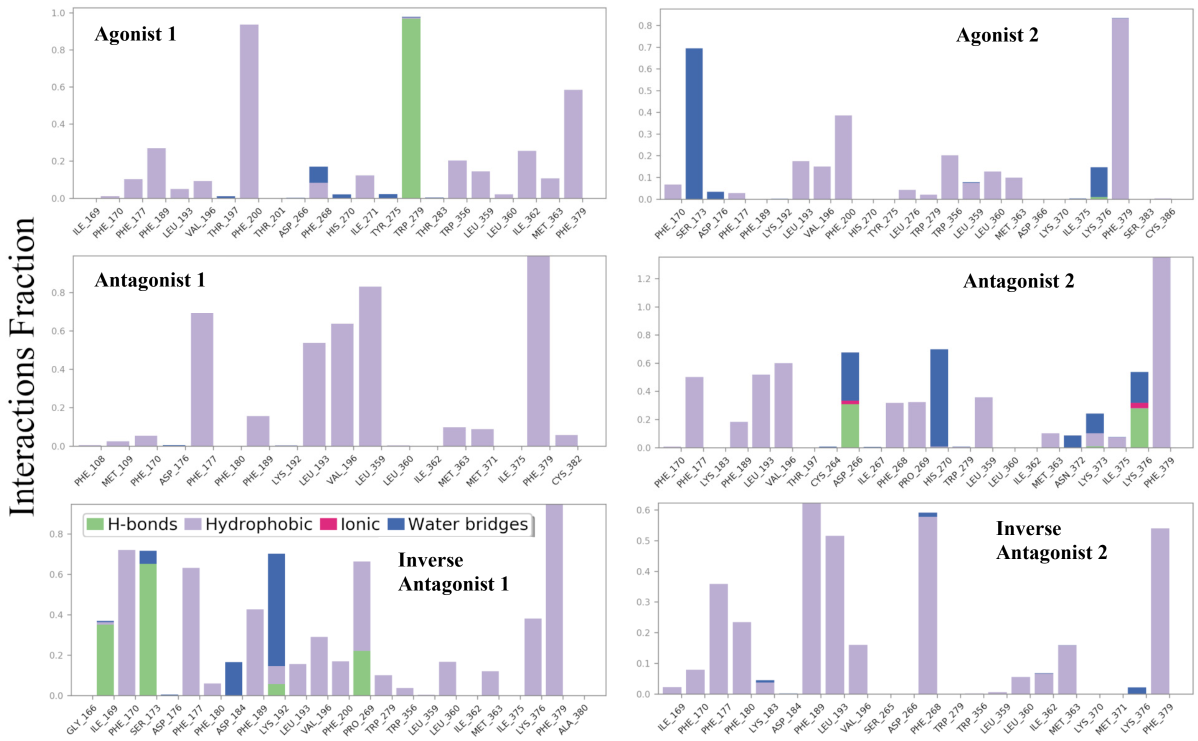

Figure S1. Interactions fraction of amino acid residues of intermediate CB_1R states during last 100ns MD simulations with agonists, antagonists, and inverse agonists.

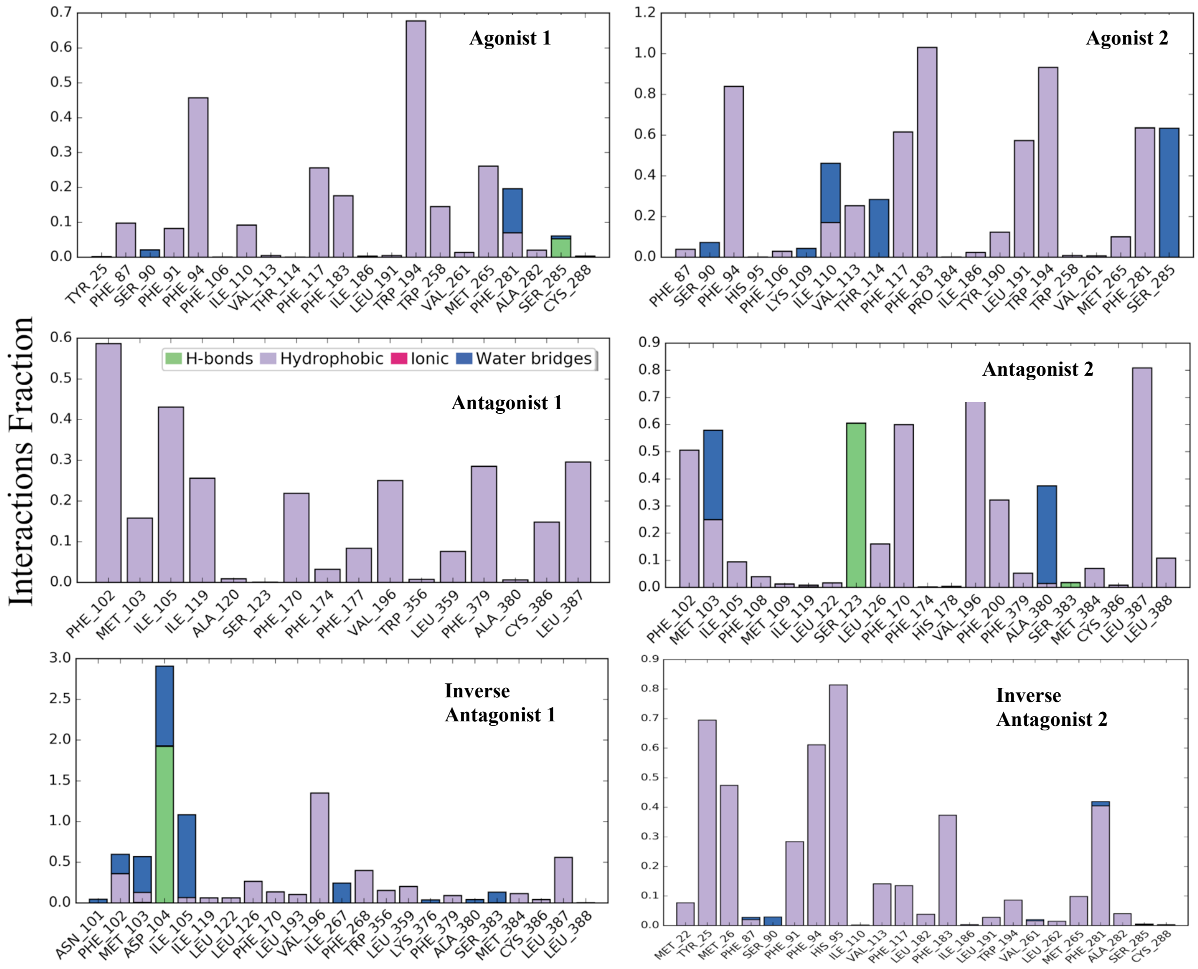

Figure S2. Interactions fraction of amino acid residues active and inactive CB2R states during last 100ns MD simulations with agonists, antagonists, and inverse agonists.

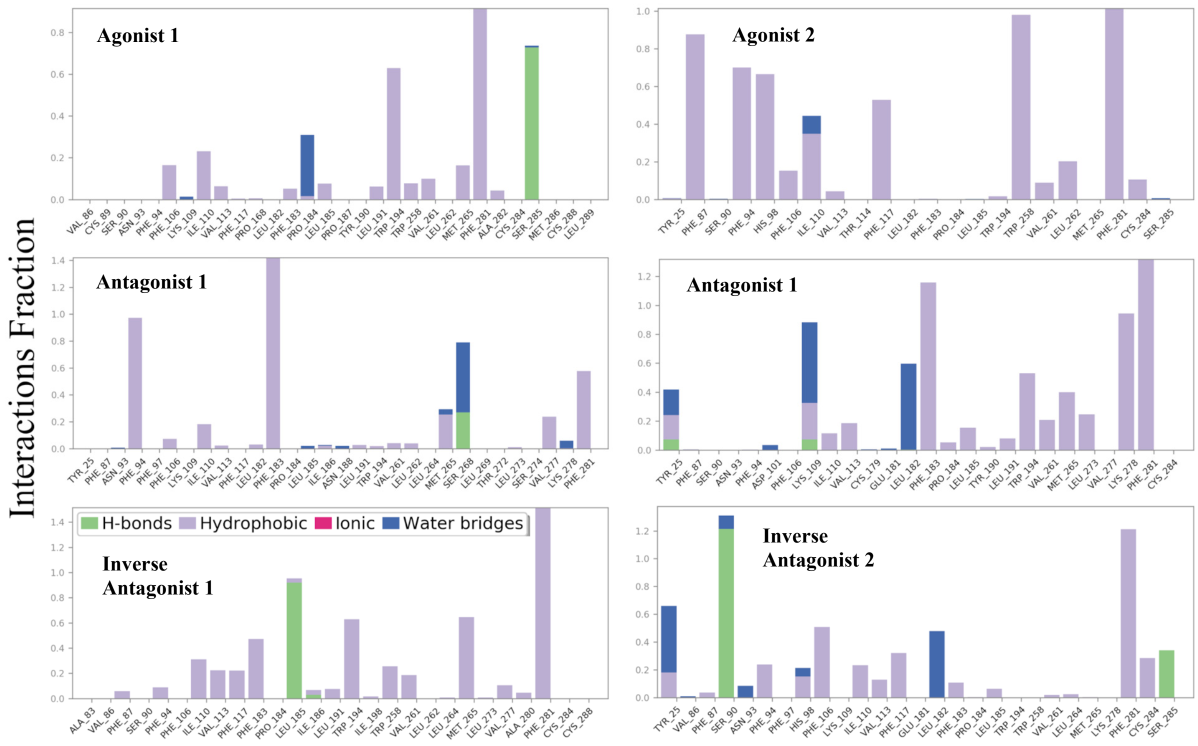

Figure S3. Interactions fraction of amino acid residues of intermediate CB2R states with agonists, antagonists, and inverse agonists.

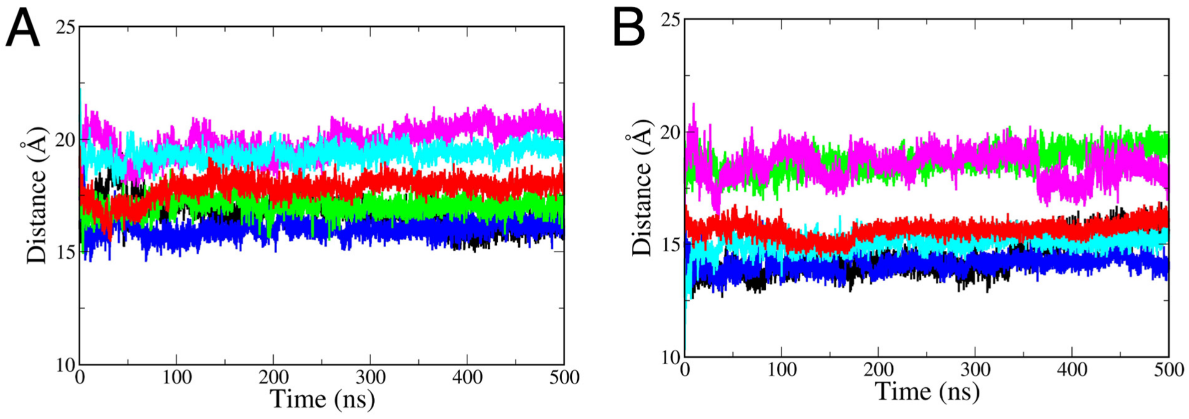

Figure S4. Locations of orthosteric binding sites in A) CB1R intermediate states and B) CB2R intermediate states.

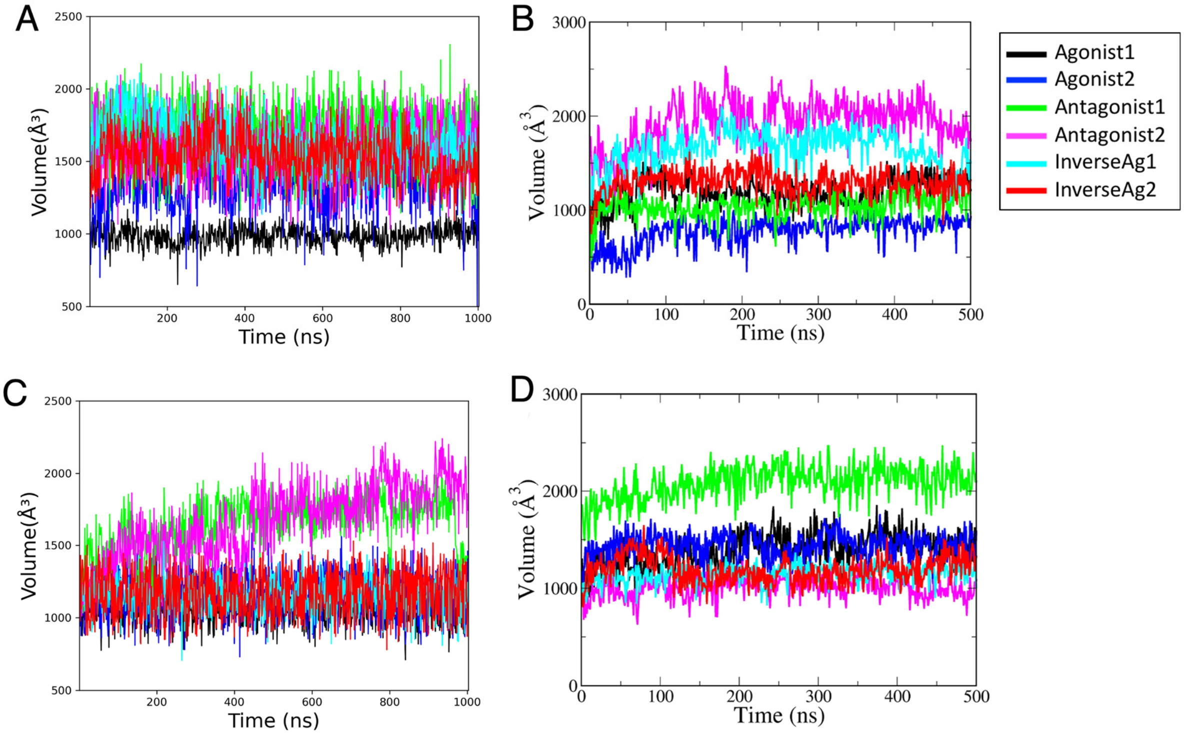

Figure S5. Volume of the binding site cavity for different ligand bound states of (A) CB_1R active and inactive states states (B) CB_1R intermediate states, (C) CB_2R active and inactive states, and (D) CB_2R intermediate states.

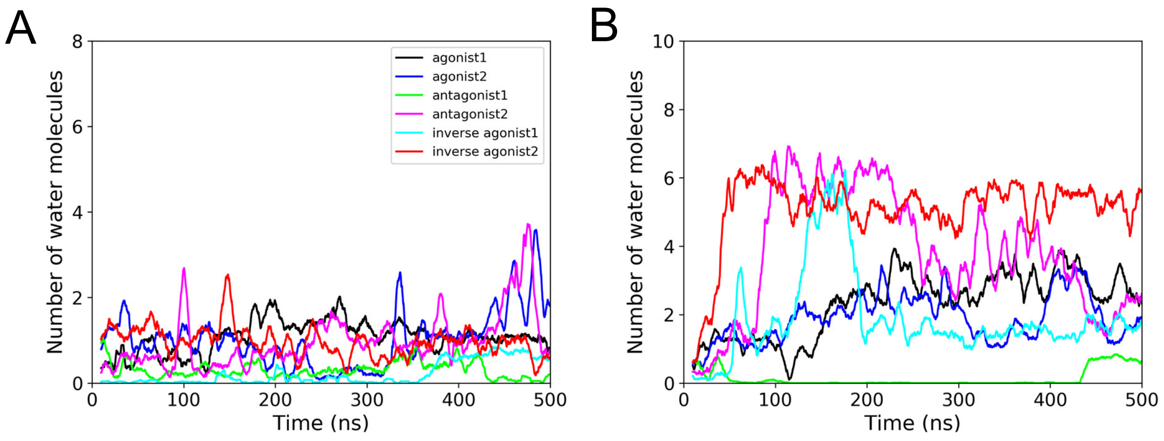

Figure S6. Number of internal waters in (A) CB_1R and (B) CB_2R intermediate states.

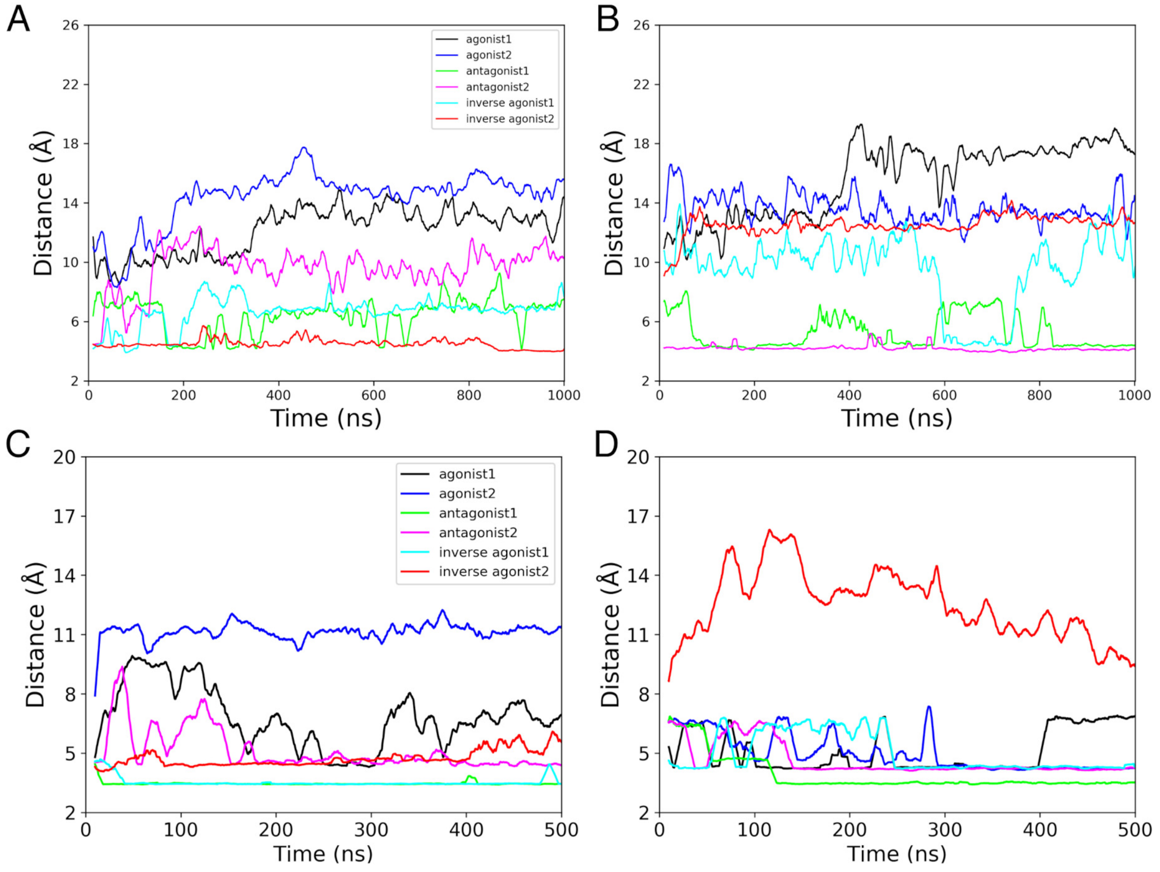

Figure S7. Running average for difference between COMs of Arg$^{3.50}$ and Asp$^{6.30}$ in (A) CB1 active and inactive states (B) CB1 intermediate states (C) CB2 active and inactive states and (D) CB2 intermediate states.

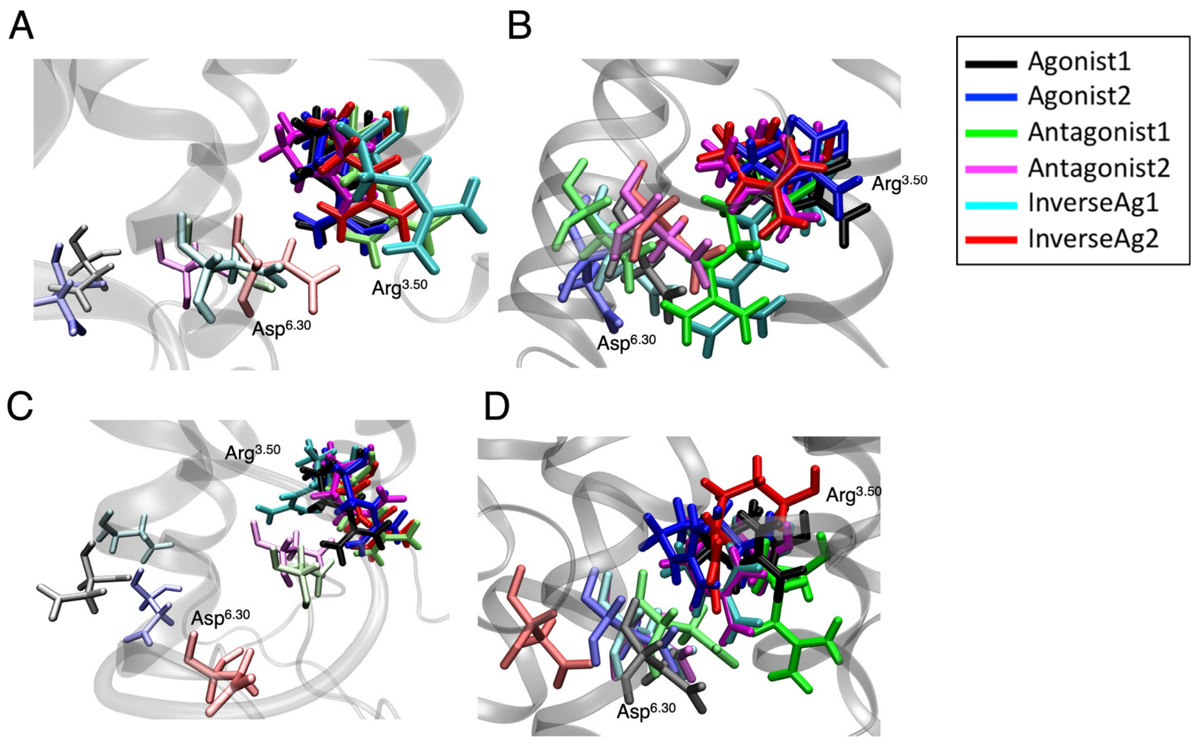

Figure S8. Positions of amino acid residues Arg$^{3.50}$ and Asp$^{6.30}$ participating in ionic lock at the end of 500ns MD simulations for (A) CB_1R regular states (B) CB_1R intermediate states, (C) CB_2R regular states, and (D) CB_2R intermediate states.

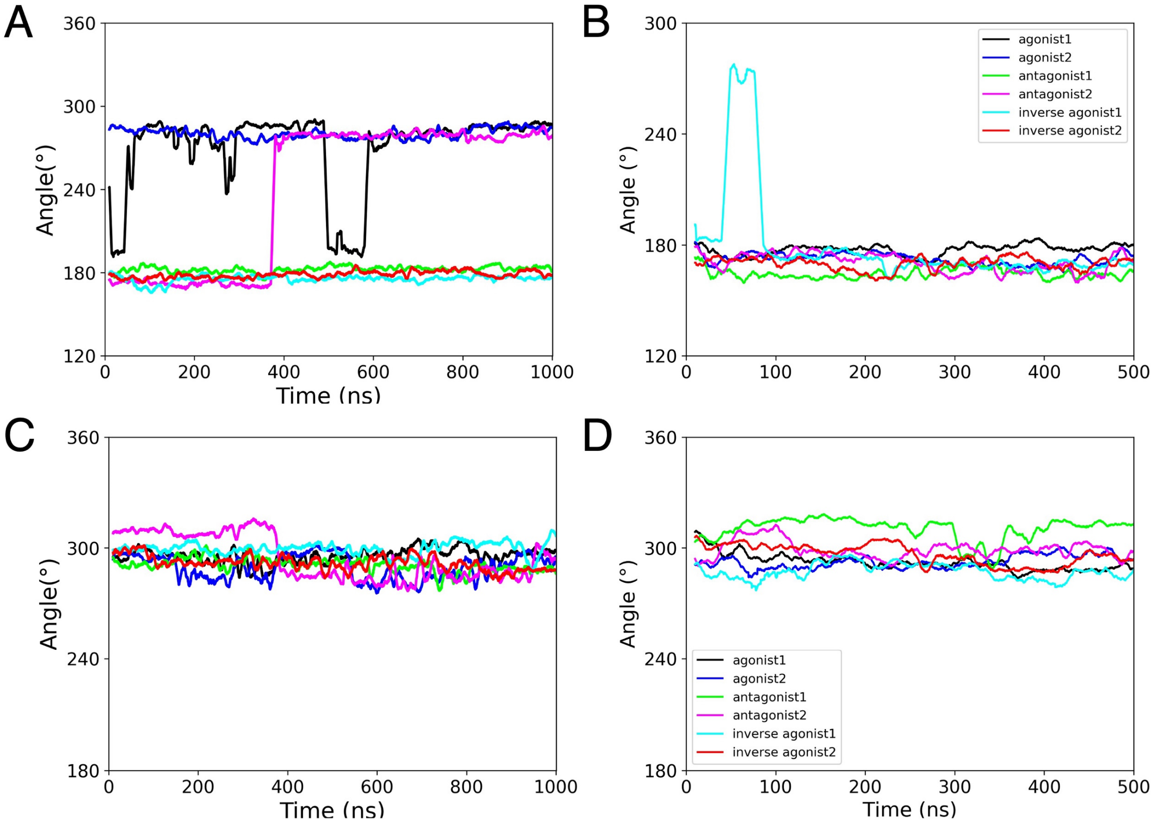

Figure S9. Dihedral angle of PHE$^{3.36}$ in (A) CB_1R active and inactive states and (B) CB_1R intermediate states; and dihedral angle of Trp$^{6.48}$ in (C) CB_1R active and inactive states and (D) CB_1R intermediate states.

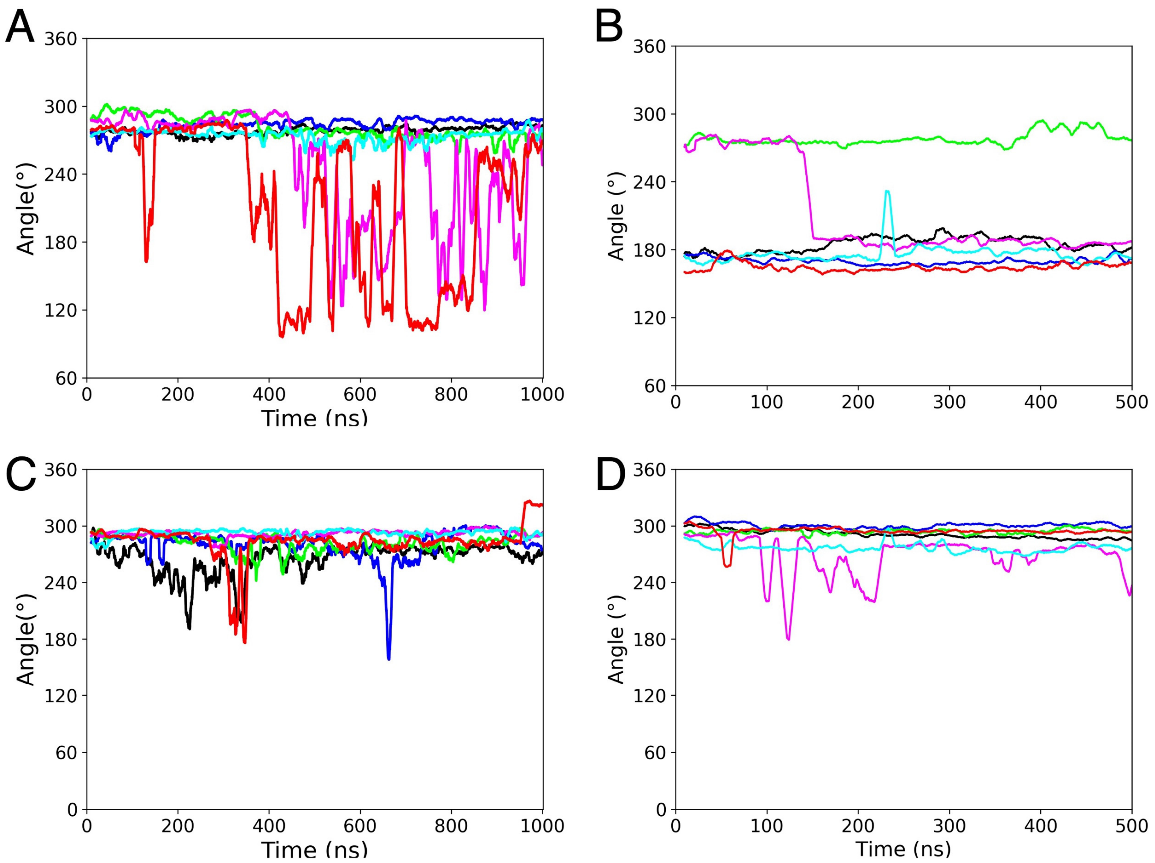

Figure S10. Dihedral angle of PHE$^{3.36}$ in (A) CB_2R active and inactive states and (B) CB_2R intermediate states; and dihedral angle of Trp$^{6.48}$ in (C) CB_2R active and inactive states and (D) CB_2R intermediate states.

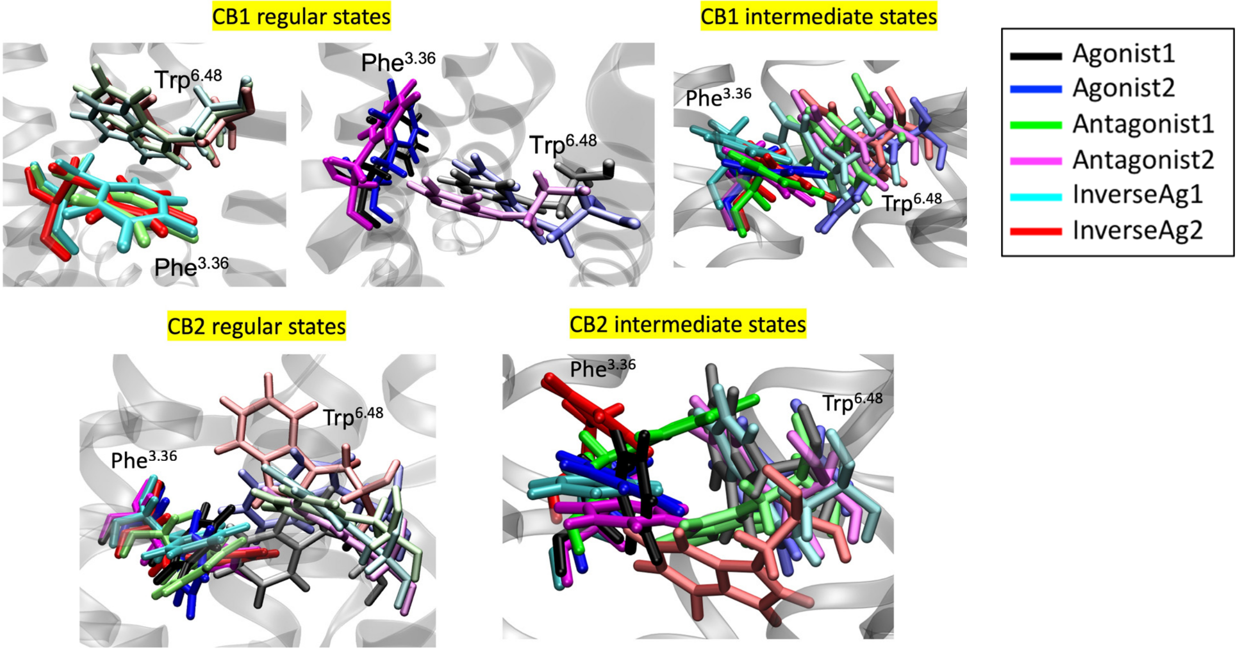

Figure S11. The orientations of Phe$^{3.36}$ and Trp$^{6.48}$ at the last MD simulation snapshot of CB_1R and CB_2R active, inactive, and intermediate states.

Systems	Simulation time (ns)	Total number of atoms	Number of water atoms
**Agonist 1**	1000	44708	25428
**Agonist 2**	1000	44641	25365
**Antagonist 1**	1000	42142	24798
**Antagonist 2**	1000	42019	24798
**Inverse Agonist 1**	1000	38233	22128
**Inverse Agonist 2**	1000	38244	22128

Table S1. System details for the active and inactive states CB_1R.

Table S2. System details for the intermediate states CB_1R.

Systems	Simulation time (ns)	Total number of atoms	Number of water molecules
**Agonist 1**	500	54743	33966
**Agonist 2**	500	54706	33933
**Antagonist 1**	500	58767	37596
**Antagonist 2**	500	58844	37662
**Inverse Agonist 1**	500	58691	37503
**Inverse Agonist 2**	500	58679	37500

Table S3. System details for the active and inactive states CB_2R.

Systems	Simulation time (ns)	Total number of atoms	Number of water molecules
**Agonist 1**	1000	38884	23103
**Agonist 2**	1000	38875	23100
**Antagonist 1**	1000	42664	24492
**Antagonist 2**	1000	42712	24537
**Inverse Agonist 1**	1000	38953	23157
**Inverse Agonist 2**	1000	38948	23148

Table S4. System details for the intermediate states CB_2R.

Syste38875ms	Simulation time (ns)	Total number of atoms	Number of water molecules
**Agonist 1**	500	48713	30084
**Agonist 2**	500	47975	29754
**Antagonist 1**	500	48258	29880
**Antagonist 2**	500	48383	29868
**Inverse Agonist 1**	500	48250	29874
**Inverse Agonist 2**	500	48924	29874
